# Micromorphology and Histology of the Secretory Apparatus of *Diospyros villosa* (L.) de Winter Leaves and Stem Bark

**DOI:** 10.3390/plants11192498

**Published:** 2022-09-23

**Authors:** Oluwatosin Temilade Adu, Yougasphree Naidoo, Temitope Samson Adu, Venkataramegowda Sivaram, Yaser Hassan Dewir, Hail Rihan

**Affiliations:** 1 School of Life Sciences, Westville Campus, University of KwaZulu-Natal, Private Bag X54001, Durban 4000, South Africa; 2 Department of Physiological Sciences, Obafemi Awolowo University, Ile-Ife 220282, Nigeria; 3 Laboratory of Biodiversity and Apiculture, Department of Botany, Bangalore University, Bangalore 560056, India; 4 Plant Production Department, College of Food and Agriculture Sciences, King Saud University, Riyadh 11451, Saudi Arabia; 5 School of Biological Sciences, Faculty of Science and Environment, University of Plymouth, Plymouth PL4 8AA, UK; 6 Phytome Life Sciences, Launceston PL15 7AB, UK

**Keywords:** histochemistry, microscopy, secretory, trichomes

## Abstract

*Diospyros villosa* is a perennial species prominently acknowledged for its local medicinal applications. The native utilisation of this species in traditional medicine may be ascribed to the presence of secretory structures and their exudate (comprised of phytochemicals). However, the morphological nature and optical features of the secretory structures in *D. villosa* remain largely unclear. This study was directed to ascertain the occurrence and adaptive features of structures found within the leaves and stem bark of *D. villosa* using light and electron microscopy techniques. The current study notes the existence of trichomes, and other secretory structures were noted. SEM indicated the presence of non-glandular hirsute trichomes with bulky stalk on both leaves and stem surfaces. Transverse stem sections revealed the existence of crystal idioblasts. Moreover, the presence of the main phytochemical groups and their localisation within the foliage and stem bark was elucidated through various histochemical tests. The trichomal length and density were also assessed in leaves at different stages of development. The results indicated that the trichomal density at different stages of development of the *D. villosa* leaves and stem bark was not significantly different from one another, F_(3,39)_ = 1.183, *p* = 0.3297. The average length of the non-glandular trichomes in the emergent, young and mature leaves, as well as in the stem, was recorded to be 230 ± 30.6 µm, 246 ± 40.32 μm, 193 ± 27.55 µm and 164 ± 18.62 µm, respectively. The perimeter and circumference of the observed trichomes in the developmental stages of *D. villosa* leaf and the stem bark were not statistically different, F_(3,39)_ = 1.092, *p* = 0.3615. The results of histochemical tests showed the existence of phenols alkaloids, which are medicinally important and beneficial for treatment of diseases. The findings of this study, being reported for the first time may be considered in establishing microscopic and pharmacognostic measure for future identification and verification of natural herbal plant. Trichomal micromorphology and histological evaluations could be utilised as a tool for appropriate description for the assessment of this species.

## 1. Introduction

The genus *Diospyros* is comprised of more than 350 species [1] and is considered most important due to its high economic value [2]. *Diospyros* is made up of shrubs and trees which are distributed across the world. Almost 42 species can be found in India, within the Central Deccan Plateau dry deciduous forest, tropical dry deciduous forest at Assam and Bengal Safari forest [3,4]. Furthermore, various species within the genus *Diospyros* are located around Africa and are highly important in both the traditional medicine and food industry [5]. *D. villosa* is a well-known species used in the maintenance of oral hygiene [6].

*D. villosa* is a plant that occurs naturally throughout the African continent [7]. *D. villosa* is a perennial, bushy, evergreen plant which could be as tall as 1–4 m. The leaves are chartaceous, dry-dull brown in colour upon on the abaxial surface and much paler on the adaxial surface. The foliage is, on average, 3 cm long, 2.5 cm wide and bovate/oblong in shape. The leaf apex is usually broadly rounded, slightly emarginated and sometimes obtuse, whereas the base is often in cordate or round shaped [6]. *D. villosa* leaves are particularly noted for having distinct hair-like epidermal structures which are referred to as ‘trichomes’. Trichomes are uni- or multi-celled structures which originate from epidermal cells of the aerial organs. These epidermal structures vary significantly in morphological characters, location, ability to secrete and type of secretion [8]. Trichomes have many functional roles within a plant. These include protection to the plant from external stress or mechanical damage [9], decreasing the heat load of a plant, maximising freezing tolerance, participation in seed dispersal, retaining water balance in plant leaves, deflection of intense radiation of the sun and protection against herbivores [10]. Additionally, the glandular trichomes offer chemical protection against different plant eating microorganisms and higher animals [11]. In addition, the exudate may be of prime importance and use in the medicinal industry

There is a lack of scientific data on the trichome morphology for many ‘*Diospyros*’ species. The morphology and structure of trichomes and the exudate found in *D. villosa* have been scarcely studied. The primary focus of this study was to investigate the histomorphology and histochemistry of the leaves and stem bark of *D. villosa*. The nature of secretory products in the leaves and stem bark were also investigated using histochemical assays in order to assess the existing constituents of the plant.

## 2. Materials and Methods

### 2.1. Plant Collection

Freshly harvested foliage and stem bark material of *D. villosa* were collected from KwaZulu-Natal, Durban, South Africa (29°84′33.6″ S, 31°4′12″ E). The plant was identified and deposited in the herbarium with number (01/18257) at the School of Life Sciences, University of KwaZulu-Natal, Durban. These samples were utilised for histological staining and morphological assessment. The developmental stages of the leaf were categorised as emergent, young and mature. A total number of ten replicates were made for each stage of the leaves and stem bark.

### 2.2. Stereomicroscopy

The structures at the top surface (abaxial) and underneath (adaxial) of the leaf and stem bark of the plant material were observed with an AZ-LED ring furnished stereomicroscope. Images were captured and processed with a Nikon AZ100 stereomicroscope furnished with a camera and the Nikon NISD Elements Software (Version 3.00, Nikon, Tokyo, Japan).

### 2.3. Electron Microscopy

Electron microscopy was used to examine the trichomal morphology in the leaf and stem of *D. villosa*. The samples were studied systemically with the aid of Nikon AZ100 stereomicroscope, Japan, attached to Nikon Fibre Illuminator and images were taken using a Nikon DXM1200C digital camera. The images were taken using the NIS Element Software.

### 2.4. Scanning Electron Microscopy

The dirt observed to have blocked the surface of the samples was washed off so as to reduce the ubiquity. Then, the leaves samples belonging to each developmental stage and the stem sections were washed with distilled water and, subsequently, with few drops of Bio-Rad tween-20 solution. The leaves and stem sections were washed once with distilled water. The sections were sectioned and fixed using glutaraldehyde (2.5%) in phosphate buffer (0.1 M, pH 7.2) for 24 h. The samples were preserved and made stable by allowing buffer washes thrice for 5 min and fixed in osmium tetroxide (0.5%) for 2 h in the absence of sunlight. Samples were later dehydrated in serial solutions (i.e., 25%, 50%, 70% and 100%). The Quorum K180 critical point dryer was used to dry the samples and later placed on aluminium stubs with the aid of carbon conductive tape and sputter-coated with gold in a QurumQ150 RES gold coater. The samples were viewed with LEO 1450 SEM (SmartSEM) and images were captured and analysed.

### 2.5. Transmission Electron Microscopy

Segments of leaf and stem tissues were excised and fixed in glutaraldehyde (2.5%) in phosphate buffer (0.1 M, pH 7.2) for 24 h. These sections were later washed thrice with buffer and fixed in osmium tetroxide (0.5%) in the absence of sunlight for 2 h. Following this, the sections were dehydrated in graded doses of acetone, i.e., 25%, 50%, 70% and 100%. The sections were infiltrated with 50% propylene oxide (50%) and Spurr’s resin for 24 h. This was further followed by allowing polymerisation of sections in 100% resin at a temperature of 85 °C for 8 h. The resin blocks were sectioned using the LKB 7801A on a Leica EM UC7 microtome (Leica Microsystems, Germany). The thinner sections were collected and stained in uranyl acetate (2.5%) for 10 min. The sections were further rinsed with slightly warm water and stained with 2.5% lead citrate and finally rinsed before viewing under a Jeol 1010 transmission electron microscope.

### 2.6. Light Microscopy

The sections from leaf and stem bark were obtained as described in the procedure for transmission electron microscopy. The Leica ultramicrotome EM UC7 (Leica Microsystems, Germany) was used for the sectioning and the sections were then stained with 1% toluidine blue for 1 min. The stained sections were viewed under a Nikon eclipse, 80i light microscope.

### 2.7. Histochemistry

The excision of sections with 100 µm thickness was made possible with the help of dental wax while using an Oxford vibratome. The obtained sections were hydrated and subsequently stained accordingly. The sections were stained with toluidine blue to detect carboxylated polysaccharides [12]. The sections were also stained with mercuric bromophenol blue to indicate total proteins [13]. Sudan back as well as Sudan IV was further used for the confirmation of total lipids and fatty acids [14]. The confirmatory test for the presence of phenolic compound was conducted using ferric trichloride [15]. The ruthenium red was used for the detection of acidic polysaccharides [14]. Meanwhile, the confirmatory test for the presence of alkaloids was performed using Wagner’s and Dittmar’s reagents [16].

### 2.8. Fluorescence Microscopy

Fresh hand cut sections of the leaf and stem bark were utilised for the purpose of this assay. The sections were mounted on the glass slide and viewed. The images were captured at various wavelengths (300 nm, 330 nm and 380 nm) using the Zeiss (Oberkochen, Germany) LSM 710 microscope, Germany. The stem bark sections were stained with 2% acridine orange for a period of 2 min. The sections were later rinsed with distilled water. The prepared sections were placed on the Zeiss LSM 710 microscope and images were captured at 488 nm. Furthermore, the obtainable leaf and stem sections were stained with Calcofluor White for 2 min and rinsed using distilled water. The sections were further placed in water and viewed using an epifluorescence microscope (Nikon Eclipse ATI) at a wavelength of 365 nm. The histomorphology of the embedded structures in the plant was conducted with the aid of Calcofluor White [17]. This stain may stain callose rather than being attached to the cellulose.

### 2.9. Energy Dispersive X-ray Microanalysis (EDX)

The elemental constituents of the leaves at developmental stages, as well as the stem bark of *D. villosa*, were determined. The elemental composition and quantification of both leaves and stem were detected by Oxford EDX detector (Oxford Instruments, Oxfordshire, UK) in a set-up of Zeiss Ultra Plus FEG-SEM (Oberkochen, Germany) at 20 kV [18]. The leaf area analyses were conducted to determine the chemical constituents of the secretory products from the plant.

### 2.10. Trichome Density, Length and Statistical Analysis

A good choice of images acquired from SEM was analysed using Image J software. The statistical package GraphPad Prism (GraphPad Software Inc., San Diego, CA, USA) was used for the data analysis. The trichomes on the leaves and stem surfaces were counted. Likewise, the trichomal density, average length, perimeter and circumference were analysed using Image J software. The quantified observations were further analysed using one-way analysis of variance (one-way ANOVA) and a Bonferroni test was used as the post-hoc analysis. The normality of data was assessed by Kolmogrov–Smirnov test and the acquired data were compared with one another using one way ANOVA when the expected requirements are duly met. *p* values less than 0.05 was standard as being significant.

## 3. Results

### 3.1. Stereomicroscopy

**Figure 1:**
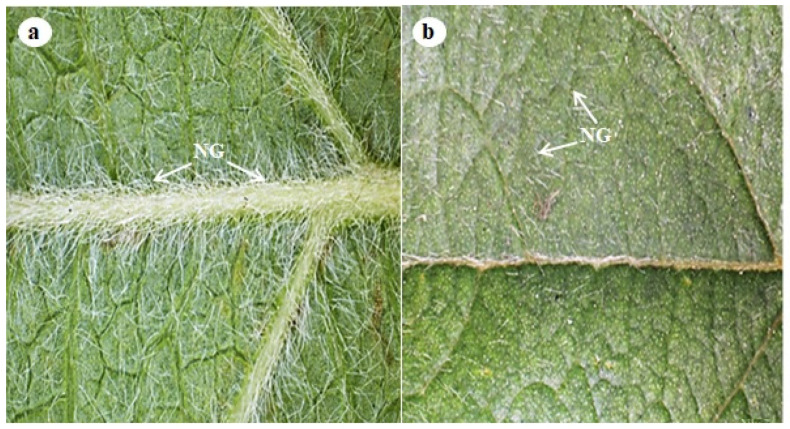
Stereomicrograph indicating the leaf topology of *D. villosa*. (**a**) Abaxial surface of the leaf with dense non-glandular trichomes along the mid and lateral veins. (**b**) Adaxial surface of the leaf showing fewer non-glandular trichomes. NG = Non glandular trichomes.

**Figure 2:**
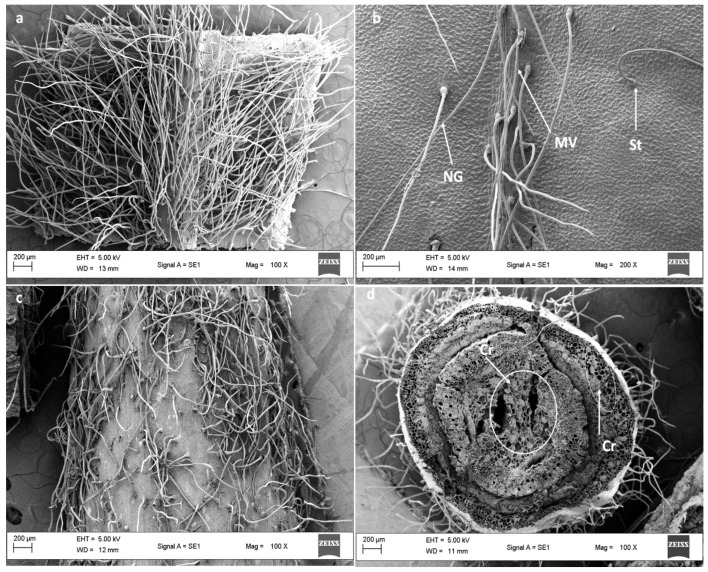
SEM micrograph of the leaf and stem bark of *D. villosa.* (**a**) Abaxial leaf surface showing the presence of numerous non-glandular trichomes across the leaf surface. (**b**) Adaxial leaf surface showing scanty non-glandular trichome coverage. (**c**) Stem surface indicating the distribution of non-glandular trichomes and (**d**) transverse sectional area of the stem bark. NG = Non glandular, MV = Medial vein, St = Stalk/base of the trichome, Cr = Crystal.

Stereomicrographs revealed that the abaxial surface of the leaf was predominantly occupied by trichomes compared with the adaxial surface (Figure 1a,b). Stereomicrographs of *D*. *villosa* revealed the sole type of trichomes (non-glandular), which was found on the stem bark (Figure 2a–d). The transverse section of *D. villosa* stem further revealed the presence of crystal idioblasts (Figure 2d).

### 3.2. Scanning Electron Microscopy

**Figure 3:**
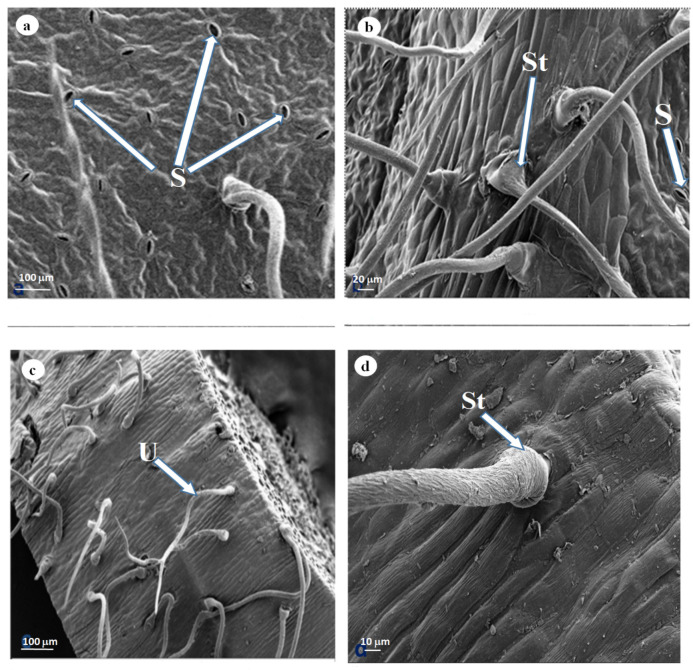
SEM of *D. villosa* leaves showing: (**a**) stomata (S) on mature leaf adaxial surface and (**b**) non-glandular single-celled stalk on the adaxial surface of mature leaf (St). SEM of *D. villosa* stem showing (**c**) non-glandular hirsute (U) trichome on surface of the stem bark and (**d**) non glandular trichome with bulky stalk (St).

Scanning electron micrographs of the leaves showed secretory pores on the epidermis (Figure 3a). Stomata/secretory pores appeared in abundance on the mature leaves’ adaxial surface compared to both the emergent and young leaves. Micrographs further revealed a single non-glandular trichome type (Figure 3c,d).

### 3.3. Transmission Electron Microscopy (TEM)

**Figure 4:**
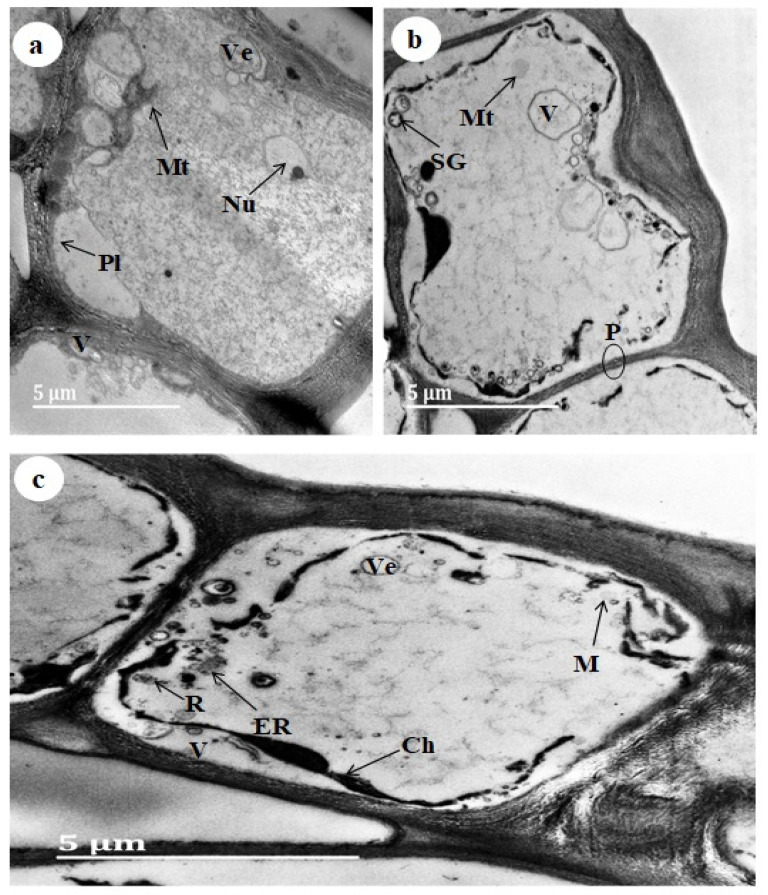
TEM micrograph of the *D. villosa* leaves at different developmental stages (**a**) Mature, (**b**) Young and (**c**) Emergent. Ch = Chloroplast, Pl = Plastids, San DiegoP = Plasmodesmata, Mt = Mitochondria, V = Vacuole, Nu = Nucleus, SG = Starch granules, ER = Endoplasmic Reticulum and R = Ribosome.

**Figure 5:**
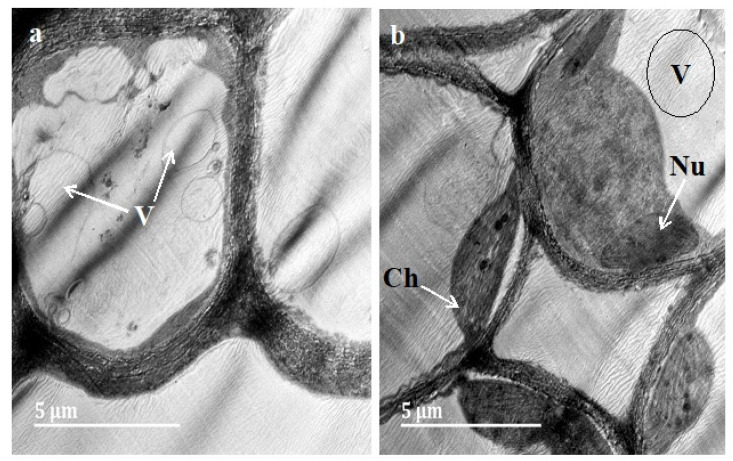
TEM micrograph of the *D. villosa* stem (**a**,**b**). Ch = Chloroplast, V = Vacuole, Nu = Nucleus.

The *D. villosa* leaves and stem bark sections were assessed using TEM. There was further observation of different molecular components, such as endoplasmic reticulum, vesicles, vacuoles (large), mitochondria, starch granule, ribosome, chloroplast and nuclei (Figure 4). The leaf was observed to consist of large vacuoles and a nucleus (Figure 4a). Moreover, plasmodesmatal connection was observed in the leaf (Figure 4b). Cytoplasm containing numerous plastids (Figure 4a) and ribosomes was also seen. Plastids were also observed to have lipophilic material, which was indicated by the presence of dark black deposits within. Furthermore, cytoplasm was seen to contain dense materials. The presence of a complex network of endoplasmic reticulum, mitochondria and plastids (Figure 4c) was further observed in the leaves. These organelles were mostly large and abundant in the cytoplasm. The vacuoles, chloroplast and vesicles were found sufficiently along the cell wall periphery (Figure 4c). Similarly, the stem was observed to be filled with large vacuoles and well observed chloroplasts (Figure 5a,b).

### 3.4. Trichome Density, Length, Perimeter and Circumference

**Figure 6:**
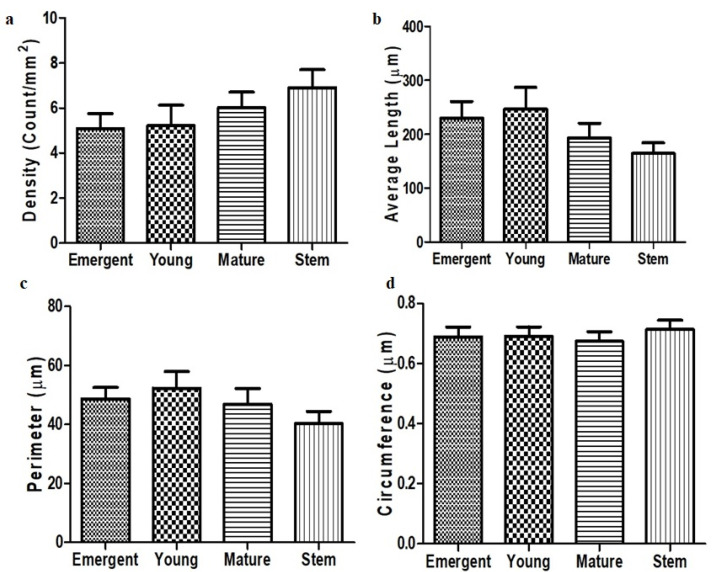
Density (**a**), average length (**b**), perimeter (**c**) and circumference (**d**) of non-glandular trichomes at different developmental stage of *D. villosa* leaves and stem bark.

Trichome density appeared to be similar across all developmental stages of the *D. villosa* leaf. Although the trichome density observed on the stem bark appeared to be higher compared to the leaf surfaces (Figure 6a), the ANOVA indicated that there were no statistical difference in the trichomal density among the developmental stages of *D. villosa* leaf and stem bark, F_(3,39)_ = 1.183, *p* = 0.3297. Similarly, the average lengths of the non-glandular trichomes were approximately 230 ± 30.6 µm, 246 ± 40.32 μm, 193 ± 27.55 µm and 164 ± 18.62 µm (Figure 6b). One-way ANOVA further showed that there was no significant difference in the average trichomal length, F_(3,39)_ = 1.478, *p* = 0.2369. The perimeter and circumference of the trichomes in the developmental stages of *D. villosa* leaf and the stem bark were not statistically different, F_(3,39)_ = 1.092, *p* = 0.3615 and F_(3,39)_ = 0.2717, *p* = 0.8454 (Figure 6c,d), respectively.

### 3.5. Histochemistry

**Figure 7:**
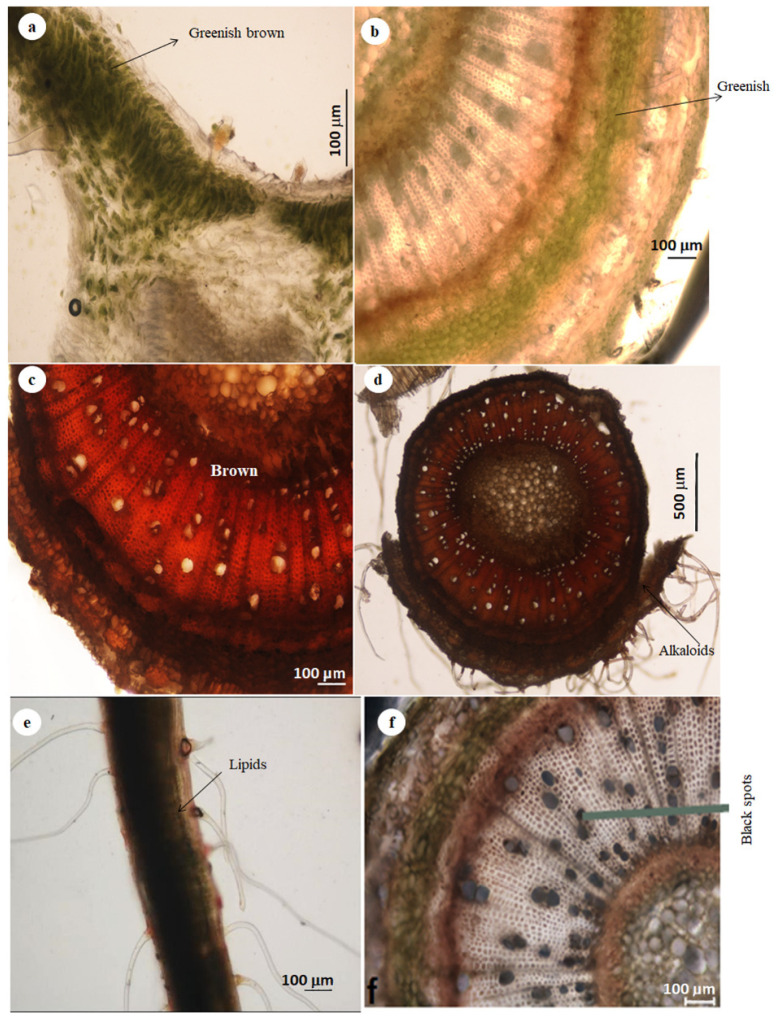
Light micrographs showing the histochemical staining characterisation of both leaf and stem sections of *D. villosa*. (**a**) Phenolic compounds stained brown on the leaf surface with Toluidine. (**b**) Phenolic compounds stained greenish brown in the stem with Toluidine. (**c**) Alkaloids stained brown colour on the *D. villosa* leaf with Dittmar reagent. (**d**) Alkaloids stained brown on *D. villosa* stem with Dittmar reagent. (**e**) Lipids stained with black stains on the leaf surface with Sudan black. (**f**) Lipids stained with black on the cross section of the stem with Sudan black.

**Figure 8:**
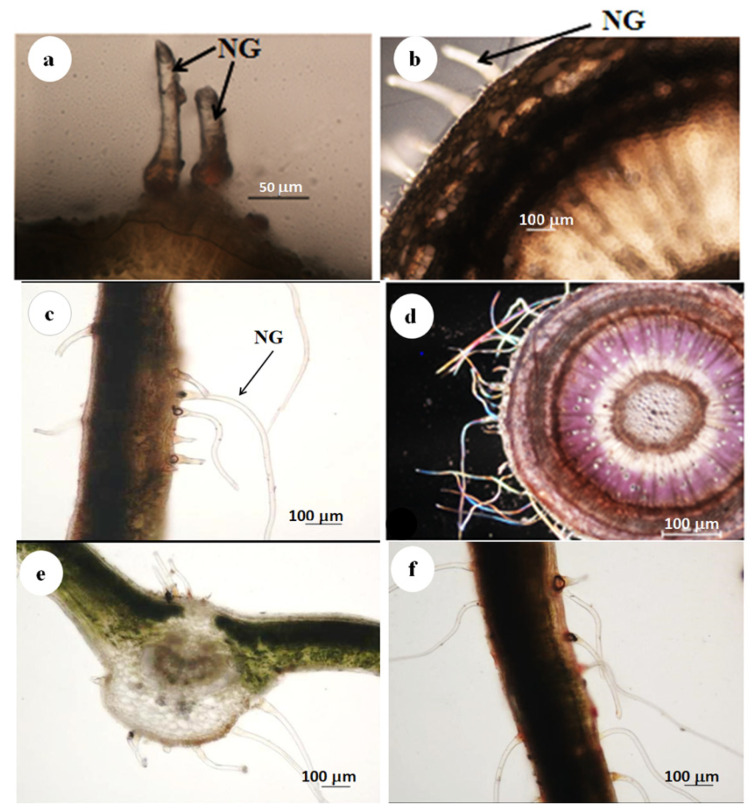
Light micrographs showing histochemical characterisation of trichomes of *D. villosa*. (**a**) Alkaloids compounds in non-glandular trichomes stained brown with Dittmar reagent. (**b**) Phenolic compounds stained red-brown in the stem and non-glandular (NG) trichomes with ferric trichloride. (**c**) Lipids stained black in the leaf and non-glandular (NG) trichomes with Nile blue. (**d**) Total protein stained purple in the stem and non-glandular trichomal section with mercuric bromophenol blue. (**e**) Lipids stained yellowish black in non-glandular trichomes with Sudan III and IV. (**f**) Acidic polysaccharides stained purplish red in non-glandular trichomes and leaf with Ruthenium red scale bar = 100 µm.

The histochemical analysis showed that lipids, phenols and alkaloids were present in the leaves and stem bark (Figure 7 and Figure 8). The greenish-brown colour further implied that the phenolic compounds (Figure 7a,b), the brown colouration as indicated in Figure 8c,d confirmed the presence of alkaloids and the black deposit indicated the presence of lipids (Figure 7e,f). These results agreed with the histochemical tests whereby various reactions indicated different colourations indicating the presence of different compounds as shown in Table 1.

### 3.6. EDX

**Figure 9:**
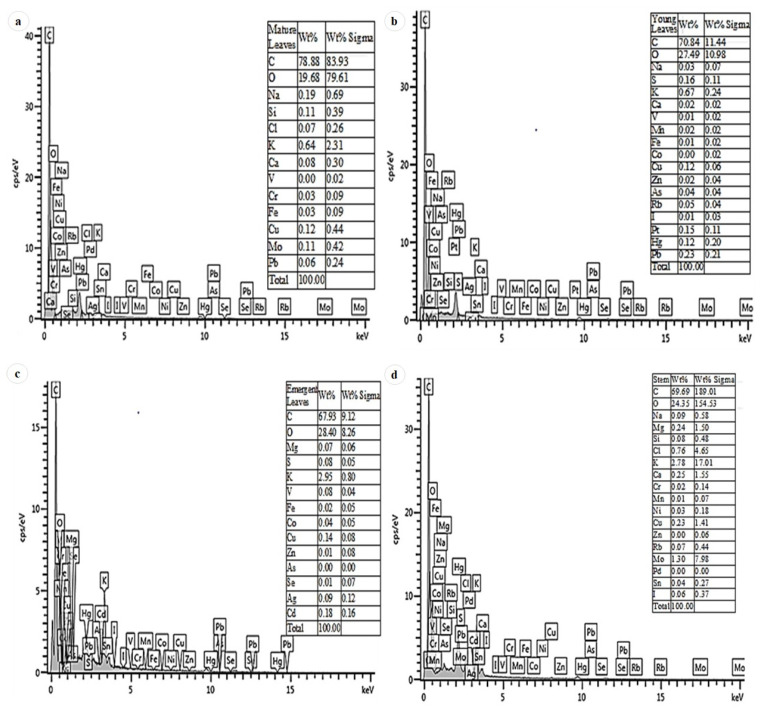
EDX spectra showing the elemental composition of the secretions of *D. villosa* leaf at the mature developmental stage (**a**), leaf at its young developmental stage (**b**), leaf at its emergent developmental stage (**c**) and stem bark (**d**).

Energy Dispersive X-ray microanalysis showed that sodium and calcium are present in the leaf sections, and the presence of sodium is observed to be higher than the calcium (Figure 9a,b). The mature leaves displayed the highest amount of sodium and calcium (Figure 9a), while the emergent leaves showed the lowest concentration of salts (Figure 9c). The sodium salts were predominantly noted within the mature leaves (Figure 9a). Similarly, the EDX spectral showed that calcium and sodium salts are present within the stem bark; meanwhile, the existence of calcium was higher compared to that of sodium (Figure 9d).

## 4. Discussion

The microscopical investigation of the leaves and stem bark of *D. villosa* revealed that the trichomes were unicellular, non-glandular and longitudinally elongated on the surface. Non glandular trichome has been previously reported for *D. sericea* and *D. hispida* [19]. However, the trichome types in *Diospyros* species were described as having unique, simple or bifurcated trichomes with walls often covered with longitudinally elongated warts and secretory cells. The microscopic analysis of trichomes in this study revealed the presence of metabolites which further served as evidence for the presence of storage cells. Trichomes are perfect storage structures for secondary metabolites and often rupture and discharge the compounds at the time of damage [20,21]. This typical attribute was noted for the *Diospyros* species, i.e., the surface of trichome tips possess globular secreting cells as well as elongated spindle-shaped warts [19]. The trichomes in *D. villosa* showed a close resemblance to species within the genus like *D. mespiliformis*, *D. lotus,* etc., and were, thus, considered to significantly regulate leaf transpiration intensity by enhancing water retention of the leaf tissues at high leaf water deficit.

The discharge exudate released through the stomata and/or secretory pores provides a defence to the leaf against herbivory grazing. Plant nitrogen is identified by an unpleasant taste [22]. This is particularly true of alkaloids, which are shown to be present on trichomes located on the leaves and stem *of D. villosa.* It may be said that the amount of exudate produced and released by the plant is comparatively related to the frequency of stomatal pores. Furthermore, the presence of star-shaped crystal idioblasts was observed in the stem sections (Figure 2d). It may be suggested that the presence of crystals in the stem sections of *D. villosa* may serve as a taxonomical informative character [23] and, perhaps a mechanism by the plant to remove excess electrolyte storage within the plant [24]. The mechanism allows identifying the crystal idioblasts which vary in shape and size. The primary function of the crystal includes promoting electrolyte homeostasis, cell support, removal of excess ions and electrolytes [25,26]. Similarly, crystal idioblasts are acknowledged to be toxic due to the embedding contents having irritating and proteolytic toxins [27], which facilitate the plant’s protection against herbivores [28]. Provided that these crystals become extremely outsized, the surrounding cytoplasmic structures may undergo lysis, which is quite dangerous for the plant [29,30]. The existence of crystal within the *D. villosa* stem may support the mechanism promoting electrolyte homeostasis in the plant.

The size of non-glandular trichomes has a significant contribution on its relative function. These trichomes serve as a mechanical impediment to pests and a defence mechanism for the plant and act as a form of physical protection to the underlying secretory cells [31]. In this study, the non-glandular trichome type accumulated phytocompounds. Hence, the trichomes also played roles in the mechanical and other measures of defence against radiation and pathogens, respectively. Similar findings were reported by Beenken [19] for *Diospyros* species. Therefore, it can be explained that trichome length varied among the families of *Diospyros* sp. and further contributed to the significance of the family. In addition, the findings of this study supported that secretions from trichomes are associated with storage cells. This was further substantiated by the histochemical analysis which indicated the presence of chemical compounds in the trichomes. Trichomes are characterised not only by their morphology, but also by their functioning (secretion, storage and mode of release). One may conclude that the trichomes of *Diospyros* differ in both morphology and physiology. EDX analyses indicated the presence of magnesium chloride in the stem bark of *D. villosa* (Figure 9d).

Examination of the morphology, distribution and the phytochemistry of the secretion associated with leaves and stem bark of a plant could further assist in elucidating possible functions of the trichomes of this plant [8,32]. The physical attributes like density, size and trichomal arrangement on the leaf surface possibly promote the protection against pests and other plant damaging organisms such as the alkaloids and phenols observed to be present in the leaves and stem bark of *D. villosa*. Non-glandular trichomes could also reduce transpiration rates and curb surface leaf exposure to intense temperatures [8,32]. The typical non-glandular trichomes observed on *D. villosa* leaves and stem bark would assume these roles as these trichomes were so dense on leaves that it is quite challenging to assess the leaf surface directly. Since *D. villosa* survive in dry territories, the observed trichomes are likely to serve a functional role in the conservation of water. This is quite similar to a study by Hameed and Hussain [33] where trichomes in plants with higher tolerance for survival in dry region were reported to enhance water conservation.

Although the non-glandular trichomes were regarded as non-secretory, the microscopy of the stained leaf section indicated that the viability of both basal and stalk cells of the trichomes (Figure 3b,d). Histochemical analysis further showed that the leaves, stem bark and trichomes of *D. villosa* accumulated the phytocompounds. Therefore, these structures played significant role in the chemical defence against insect, herbivores and pathogens. The prominent chemical compounds responsible for these functional roles are phenols and alkaloids. These phytochemical compounds, in accordance with Soni et al. [34], were of medicinal importance and repute. Alkaloids are nitrogenous chemical compounds which were reported to treat different ailments and diseases like inflammation [35], oxidative stress and inflammation [36], asthma and fever [37]. Alkaloids appeared as active metabolites and natural repellents against insect herbivores and natural enemies [38,39]. In addition, phenolics were classified as abundant secondary metabolites [40]. The phenolic compounds functioned adequately against pests and pathogens. Upon the release of phenols, they are further oxidised to quinones by polyphenol oxidase, thus facilitating the entrapment of insect on the leaf surface. The presence of phenolic compounds within the trichomes (Figure 8a,b) explained the insect entrapment ability of *D. villosa.* Moreover, phenolics promoted the plant’s defence against pathogens and ultraviolet radiations [41]. These compounds were abundant in all plant segments and contained potent antioxidant activities in comparison with other curative and pharmaceutical uses and are used in aesthetic and lumbering industry [42].

Previous reports highlighted the importance of micromorphological and histological screening of medicinal plants, which greatly helps the researchers in the field to develop further research studies on medicinal plants in their respective field. The findings of the present study will be used to supplement pharmacognostical evaluations, correct identification and verification of this plant species. The present work on *D. villosa* will also provide a basic knowledge to the researchers for further studies on effect of phytocompounds detected, characterisation and significance of other special microscopic features that can be utilised as a measure for natural medicinal plant.

It is concluded that the emanating study provided novel information regarding the micromorphology and functions of microstructures of *D. villosa* leaves and stem bark. Non-glandular trichomes were observed on both leaves and stem bark of *D. villosa*. The histochemical analysis further indicated the deposition of alkaloids and phenolic compounds in the leaves and stem bark of *D. villosa*. These compounds are of ecological and medicinal importance as they have a chemical defence mechanism against pathogens and are of use in the medicinal industry in treating a range of ailments. However, secretory products should be further evaluated and a comprehensive phytochemical screening should be conducted so as to establish all other phytochemicals in the plants.

## Figures and Tables

**Table 1:** Observations of histochemical tests on fresh leaf and stem bark sections of *D. villosa*.

Compounds	Stains	Leaves/Stem	Trichomes	Reactions Observed
Alkaloids	Dittmar’s	+	+	Brownish colouration in the stem as well as the trichomes
Lipids	Sudan III and IV	+	+	Cells in the leaf and stem sections stained black, the trichomes stained black as well
	Nile Blue	+	+	Black colouration in the leaf sections and non-glandular trichomes
Phenols	Ferric trichloride	+	+	Brown deposits on the cells of the leaf sections, the non-glandular cells further stained brown
Acidic Polysaccharides	Ruthenium red	+	+	Leaf and non-glandular trichomes stained purplish red
Total protein	Mercuric bromophenol blue	+	+	Stem cells and non-glandular trichomes stained purple
Polyphenols (lignin, tannins)	Toluidine blue	+	+	Leaf cells and non-glandular trichomes stained brown

(+) indicates presence of compounds.

## Data Availability

Not applicable.
